# Inflammatory biomarkers and risk factors in depression: a longitudinal study in adults

**DOI:** 10.1192/j.eurpsy.2025.1308

**Published:** 2025-08-26

**Authors:** G. Cano-Escalera, I. Gómez Grávalos, M. Melero González, I. Zorrilla, P. Lopez Pena, K. S. MacDowel, J. C. Leza, A. Gonzalez-Pinto

**Affiliations:** 1 CIBER de Salud Mental, Instituto de Salud Carlos III, Madrid; 2 Department of Psychiatry, Araba University Hospital; 3 Bioaraba Health Research Institute, BIOARABA, Vitoria; 4 Department of Computer Science and Artificial Intelligence, Computational Intelligence Group, Donostia; 5 Department of Pharmacology and Toxicology, Facultad de Medicina, IIS Hospital 12 de Octubre, IUIN-UCM, Madrid, Spain

## Abstract

**Introduction:**

Depression is a chronic and recurrent mental condition, causing a high burden of disease, functional impairment, and significant economic costs worldwide.

**Objectives:**

To compare the neuropsychological profile, inflammation, and functionality in patients with a major depressive episode before and after treatment.

**Methods:**

A longitudinal study was conducted, recruiting a total of 39 subjects, with a mean age of 42 years (12 men - 30.8% and 27 women - 69.2%) diagnosed with major depressive disorder (MDD). The neuropsychological profile was measured using the Screening of Cognitive Impairment in Psychiatry (SCIP) and the level of functionality with the Brief Assessment of Functioning (FAST) test. Both total scores and subscales were used, along with inflammatory biomarkers (IL-6, IL-1ß, TNF-α, PGE2) and oxidative stress markers (TAS, GSHtot, GSHfree, GSSG, CAT, SOD, GPx, and BDNF).

**Statistical analysis:**

The paired t-test was used through the statistical program SPSS. A unilateral p-value less than 0.05 was considered statistically significant.

**Results:**

Significant differences were observed in both the Hamilton Depression Rating Scale (p < 0.001) and in the biomarkers total GSH (p < 0.15), GSH free (p < 0.029), and GSSG (p < 0.027) of patients at three months from the start of pharmacological treatment compared to baseline (Table 1). Differences were also observed at 3 months of treatment in the SCIP scale (p < 0.001) as well as in its subscales: Immediate verbal learning (p < 0.001), Working memory (p < 0.001), Verbal fluency (p = 0.007), and Delayed verbal learning (p < 0.001), and in the FAST scale (p < 0.001) and its subscales: Autonomy (p = 0.002), Cognitive (p = 0.004), Finances (p = 0.004), and Relationships (p < 0.001) (Table 2).

**Image 1:**

**Figure:**
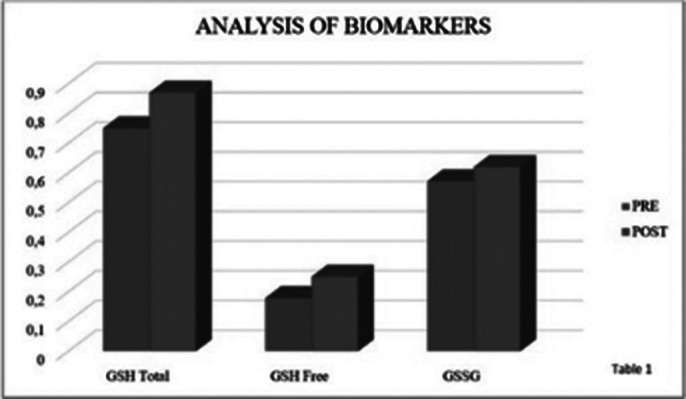

**Image 2:**

**Figure:**
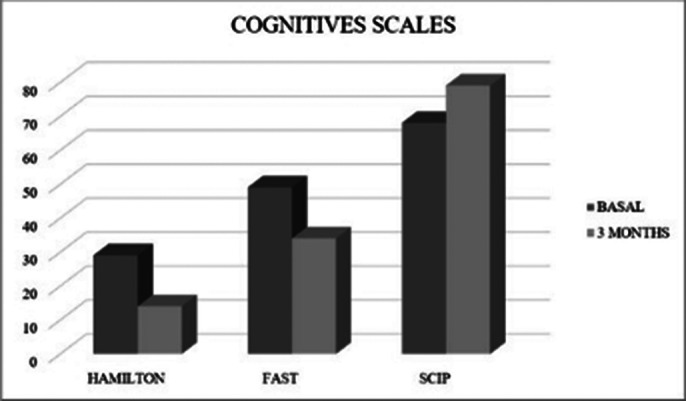

**Conclusions:**

Our results indicate that there are improvements at the cognitive and functional level in subjects taking antidepressant treatment. Changes in GSH and GSSG biomarkers were also observed, whose antioxidant function is crucial for protecting cells from oxidative damage. The findings suggest that oxidative stress is a crucial factor in the pathophysiology of MDD. The study highlights the potential of these biomarkers to guide therapeutic strategies and improve the management of depression.

**Disclosure of Interest:**

None Declared

